# Triggers and oncologic outcome of salvage radical prostatectomy, salvage radiotherapy and active surveillance after focal therapy of prostate cancer

**DOI:** 10.1007/s00345-021-03700-x

**Published:** 2021-04-21

**Authors:** Jost von Hardenberg, Hannes Cash, Daniel Koch, Angelika Borkowetz, Johannes Bruendl, Sami-Ramzi Leyh-Bannurah, Timur H. Kuru, Karl-Friedrich Kowalewski, Daniel Schindele, Katharina S. Mala, Niklas Westhoff, Andreas Blana, Martin Schostak, Working Group of Focal and Microtherapy of the German Society of Urology (DGU)

**Affiliations:** 1 Department of Urology and Urosurgery, University Medical Centre Mannheim, Medical Faculty Mannheim, University of Heidelberg, Theodor-Kutzer-Ufer 1-3, 68167 Mannheim, Germany; 2 Department of Urology, Charité University Medicine Berlin, Berlin, Germany; 3 PROURO, Berlin, Germany; 4 Clinic of Urology, Urooncology, Robotic-Assisted and Focal Therapy, Medical Faculty and University Clinics of Magdeburg, Magdeburg, Germany; 5 Department of Urology, Fuerth Hospital, Fuerth, Germany; 6 Department of Urology, University Hospital Carl Gustav Carus, Technische Universität Dresden, Dresden, Germany; 7 Department of Urology, Caritas St. Josef Medical Center, University of Regensburg, Regensburg, Germany; 8 Department of Urology, Pediatric Urology and Urologic Oncology, Prostate Center Northwest, St. Antonius-Hospital, Gronau, Germany; 9 Department of Urology, University Hospital Cologne, University of Cologne, Cologne, Germany

**Keywords:** Prostate neoplasms, Salvage therapy, Focal therapy, Partial gland ablation, Hemi-ablation, High-intensity focused ultrasound, Multiparametric magnetic resonance imaging

## Abstract

**Purpose:**

Due to the tissue preserving approach of focal therapy (FT), local cancer relapse can occur. Uncertainty exists regarding triggers and outcome of salvage strategies.

**Methods:**

Patients with biopsy-proven prostate cancer (PCa) after FT for localized PCa from 2011 to 2020 at eight tertiary referral hospitals in Germany that underwent salvage radical prostatectomy (S-RP), salvage radiotherapy (S-RT) or active surveillance (AS) were reported. Prostate specific antigen (PSA) changes, suspicious lesions on mpMRI and histopathological findings on biopsy were analyzed. A multivariable regression model was created for adverse pathological findings (APF) at S-RP specimen. Kaplan–Meier curves were generated to determine oncological outcomes.

**Results:**

A total of 90 men were included. Cancer relapse after FT was detected at a median of 12 months (IQR 9–16). Of 50 men initially under AS 13 received S-RP or S-RT. In total, 44 men underwent S-RP and 13 S-RT. At cancer relapse 17 men (38.6%) in the S-RP group [S-RT *n* = 4 (30.8%); AS *n* = 3 (6%)] had ISUP > 2. APF (pT ≥ 3, ISUP ≥ 3, pN + or R1) were observed in 23 men (52.3%). A higher ISUP on biopsy was associated with APF [*p* = 0.006 (HR 2.32, 97.5% CI 1.35–4.59)] on univariable analysis. Progression-free survival was 80.4% after S-RP and 100% after S-RT at 3 years. Secondary therapy-free survival was 41.7% at 3 years in men undergoing AS. Metastasis-free survival was 80% at 5 years for the whole cohort.

**Conclusion:**

With early detection of cancer relapse after FT S-RP and S-RT provide sufficient oncologic control at short to intermediate follow-up. After AS, a high secondary-therapy rate was observed.

**Supplementary Information:**

The online version contains supplementary material available at 10.1007/s00345-021-03700-x.

## Introduction

Localized prostate cancer (PCa) is a heterogenous disease affecting one out of nine men during their lifetime in Germany [1]. Focal therapy is emerging as a potential additional therapy option in highly selected patients with localized PCa [2]. Between 12.8 and 16.2% patients with fusion-biopsy-proven PCa potentially qualify for focal therapy (FT) according to international consensus statements [3]. Despite a comprehensive diagnostic pathway for patient selection before FT, local cancer relapse after FT can occur within or out of the ablation zone due to the tissue preserving strategy and potential ablation energy failure [4, 5]. Salvage radical prostatectomy (S-RP) has been reported as feasible but could lead to inferior oncological outcomes compared to primary radical prostatectomy [6]. Salvage-radiotherapy (S-RT) has only been described in one report [7]. Active surveillance (AS) and comparative reports in this setting have not been described hampering decision-making and exposing patients potentially to overtreatment and undertreatment.

We undertook a multi-center retrospective study to assess triggers for different salvage therapies, determine predictors of adverse pathological findings (APF) at S-RP specimen and assess oncological outcomes across all three salvage-strategies. The analysis helps clinicians with decision-making in case of cancer relapse after FT and prior FT in context of the duty to fully inform patients about this alternative therapy.

## Patients and methods

### Patient population

Patients after FT for localized PCa who underwent control biopsies (per protocol within clinical trials or due to suspected PCa relapse) that confirmed cancer relapse after FT at eight tertiary referral centers in Germany were included in the study. Patients must have undergone FT in a true focal approach or hemiablation. All energy sources were allowed for inclusion, but most FT are performed by high intensity focused ultrasound (HIFU) in Germany. Localization of FT failure was approximated based on biopsy documentation and FT treatment localization if information was available. Patients with subsequent S-RP, S-RT or AS were included into the analysis. AS was defined as a minimum of 6 months without further treatment after detection of cancer relapse after FT. All participating centers are German Cancer Society (Deutsche Krebsgesellschaft, DKG)-certified prostate cancer centers (ecc-cert.org). Within this framework, biopsies and S-RP specimens were reported. As most patients were treated within clinical FT trials, most of the data were prospectively collected for the retrospective analysis.

### Triggers and outcomes

MRI/TRUS-fusion biopsies were performed on different platforms according to the internal protocol of each center. MRI images were read and interpreted according to PI-RADS [8]. PSA measurements were routinely performed before interventions, in between and thereafter according to the treating urologists. PSA change in percent was calculated for the individual patient if PSA data were available. Adverse pathological findings at S-RP were defined as pT ≥ 3a, pN+, ISUP ≥ 3 and/or R1. Progression-free survival (PFS) was defined as the time from salvage therapy to biochemical [S-RP: PSA ≥ 0.2 ng/ml; S-RT: PSA nadir + 2 ng/ml (Phoenix criteria)] or radiologic progression. Secondary therapy-free survival was defined as the time from detection of cancer relapse after FT to further treatment (S-RP, S-RT, re-do FT or androgen deprivation therapy). Metastasis-free survival was defined as the time from cancer relapse after FT to the detection of distant metastases or histological proven lymph-node metastases at lymphadenectomy specimen.

### Statistical analysis

Absolute and relative frequencies were assessed for categorical variables, while means and standard deviation (SD) as well as medians and interquartile ranges (IQR) were computed for continuously variables. To compare groups repeated measurements ANOVA was performed, in case of significance a post hoc test using the Turkey’s multiple comparisons test was calculated. To identify potential influential factors for the prediction of APF at S-RP specimens univariable analysis was performed. Independent variables with a *p* value of ≤ 0.2 were included in multivarible logistic regression analyses. All test were two-sided with a statistical significance set at *p* ≤ 0.05. Analyses were conducted using R (R Core Team (2019) R: a language and environment for statistical computing. R Foundation for Statistical Computing, Vienna, Austria) or Graph Pad Prism Version 9 (San Diega, CA, US).

## Results

Of the 98 men enrolled with proven cancer relapse after FT between 2011 and 2020, 90 received the subsequent salvage strategy of interest: S-RP, S-RT or AS (CONSORT diagramm see online resource). FT was performed in these patients using HIFU [*n* = 88 (97.8%)] or focal vascular targeted therapy [*n* = 2 (2.2%)]. The distribution of FT strategy is depicted in Table 1. FT failure was localized in the treated field in 24 (54.6%) patients in the S-RP group [out of field 6 (13.6%); in and out of field 1 (2.7%); not assignable 13 (29.6%)]. FT failure occurred in the treated field in 9 patients (69.2%) in the S-RT group [out of field 2 (15.4%); not assignable 2 (15.4%)]. FT failure was observed in-field in 24 patients (48%) in the AS group [out of field 13 (26%); in and out of field 2 (4%); not assignable 11 (22%)]. S-RP was performed open (*n* = 16), conventional laparoscopic (*n* = 3) or robot-assisted laparoscopic (*n* = 25). S-RT was delivered via image-guided RT conventional fractionation in 12 patients (range 74–80 Gy, 2 Gy/fraction) and moderate hypofractionation in one patient (60 Gy, 3 Gy/fraction). At least six months combined androgen deprivation therapy (ADT) was administered to six patients (46.2%; 6 patients: no ADT, one patient: unknown). The D´Amico risk group distribution of all patients before FT and at cancer relapse after FT is shown in the online resource Table 1.

**Table 1:** Patient characteristics and perioperative data

Variable	S-RP *n* = 44	AS *n* = 50	S-RT *n* = 13
	Value		
Mean ± SD age at cancer relapse or salvage therapy (ST)	65.1 ± 8.0	68.2 ± 7.6	73.9 ± 7.2
Median PSA ng/ml at cancer relapse or ST (IQR)	5.7 (3.8–9.1)	3.48 (2.1–5.4)	8.6 (5.2–11.9)
No. biopsy strategy at cancer relapse (%)			
Systematic only	4 (9.1)	7 (14)	1 (7.7)
Targeted only	4 (9.1)	1 (2)	–
Combined systematic and targeted	32 (72.7)	42 (84)	11 (84.6)
No. ISUP GG at cancer relapse (%)			
GG 1	17 (38.6)	42 (84)	4 (30.8)
GG 2	10 (22.7)	5 (10)	4 (30.8)
GG ≥ 3	17 (38.6)	3 (6)	4 (30.8)
Median % sys biopsy cores pos at cancer relapse (IQR)	16.7 (9.6–35)	8.7 (8.3–16.7)	23.3 (8.3–36.1)
Median max. infiltration (%) of cores (IQR) at cancer relapse	30 (17.5–52.5)	15 (10–30)	30 (17.5–62.5)
No. PI-RADS at cancer relapse (%)			
PI-RADS 3	2 (4.5)	3 (6)	–
PI-RADS 4–5	19 (43.2)	14 (28)	1 (7.7)
Suspicious, no PI-RADS	1 (2.3)	4 (8)	–
Not suspicious	16 (36.4)	29 (58)	11 (84.6)
No. focal therapy (FT) energy source			
High-intensity focused ultrasound	42 (95.5)	50 (100)	13 (100)
Vascular targeted therapy	2 (4.5)	–	–
No. previous FT strategy (%)			
Focal	25 (56.8)	35 (70)	11 (84.6)
Hemi-ablation	14 (31.8)	11 (22)	1 (7.7)
Focal strategy other/unknown	5 (11.4)	4 (8)	1 (7.7)
Median PSA change (%) from FT to nadir (IQR)	− 47.5 (− 62.2 to 27.6)	− 58.5 (− 75.8 to 40.7)	− 54.0 (− 87.1 to 41.0)
Median PSA change (%) from FT to cancer relapse or ST (IQR)	− 20.6 (− 38.7 to 0.5)	− 50.1 (− 70.0 to − 34.2)	18.8 (− 24.2 to 55.9)
Median PSA change (%) from nadir to cancer relapse or ST (IQR)	40.1 (13.3 to 103.4)	19.1 (0 to 38.6)	125.0 (52.8 to 571)
Median months from FT to cancer relapse (IQR)	11 (8.75–17)	12 (11–15)	12 (12–14.5)
Median months follow-up after cancer relapse (IQR)	28 (15 to 44.5)	19 (8.3 to 36)	34.5 (26.3 to 46.3)

### Triggers for choice of subsequent therapy

Table 1 summarizes the clinico-pathological characteristics of the cohort and potential triggers for the choice of subsequent salvage therapy. Mean (SD) patient age was 65 years (± 8.0) in the S-RP group [S-RT group: 73.9 (± 7.2); AS group: 68.2 (± 7.6)]. Median (IQR) PSA at cancer relapse after FT was 5.7 ng/ml (3.8–9.1) in the S-RP group [S-RT group: 8.6 (5.2–11.9); AS group: 3.48 (2.1–5.4)]. Most patients across all groups had ISUP 1 or 2 disease at cancer relapse after FT with a median of < 20% of cancer positive systematic biopsy cores.

**Fig. 1:**
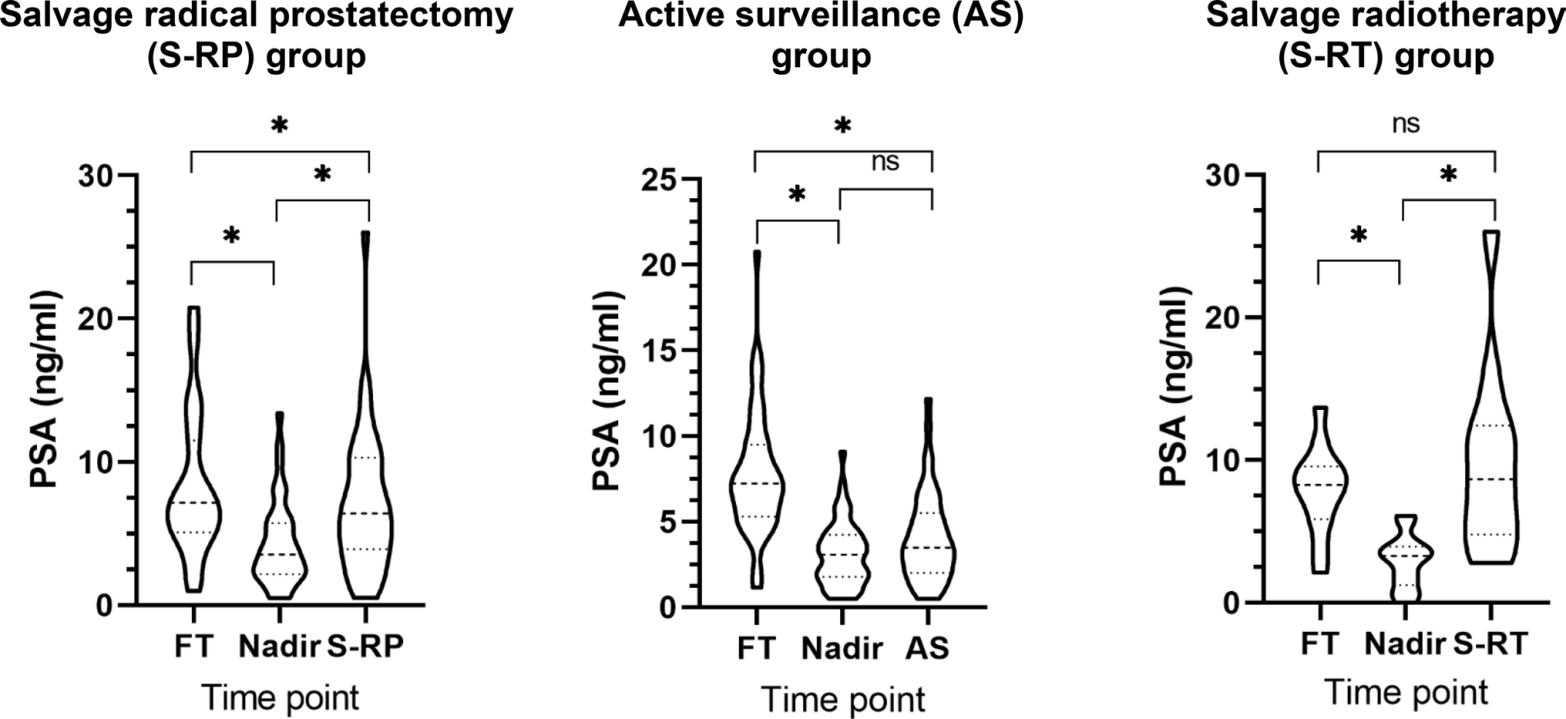
PSA changes as trigger for salvage therapy after cancer relapse. *p ≤ 0.05. *ns* not significant

The violine plots (Fig. 1) illustrate the comparisons of PSA values at FT, PSA nadir post FT and initiation of salvage therapy in all three groups. The PSA values in the AS group did not differ significantly between PSA nadir and initiation of AS. Median (IQR) PSA levels in the S-RP group at FT were 7.2 ng/ml (5.1–11.5) and at nadir 3.5 ng/ml (2.2–5.7), in the AS group at FT 7.2 ng/ml (5.3–9.5) and at nadir 3.1 ng/ml (1.8–4.2) and in the S-RT group at FT 8.3 ng/ml (5.9–9.6) and at nadir 3.3 ng/ml (1.2–3.9).

### Adverse pathological findings

**Fig. 2:**
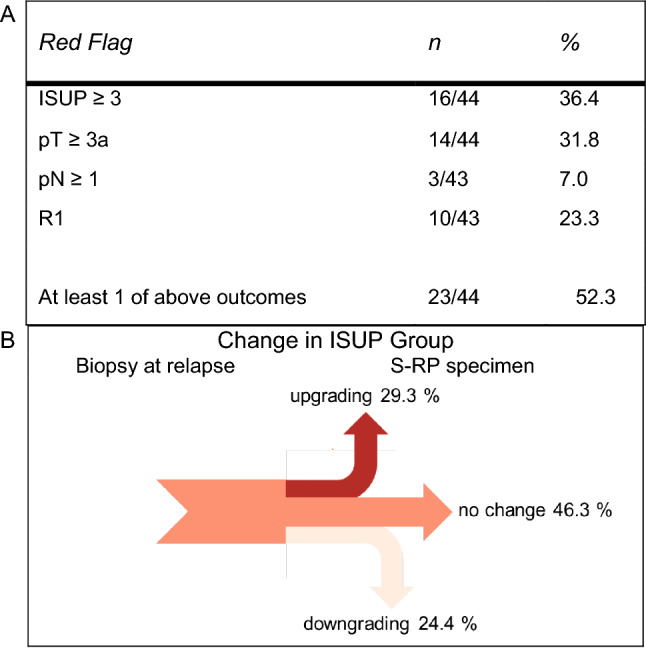
**a** Adverse pathological findings on S-RP, **b** Sankey diagram emphasizes proportions of ISUP changes between biopsy at relapse compared to S-RP specimen

Figure 2a summarizes patients with APF at S-RP. APF were observed in 23 men (52.3%). Only ISUP at cancer relapse after FT was associated with APF [*p* = 0.006 (HR 2.32, 95% CI 1.35–4.59)] on univariable analysis (see online resource Table 2). No predictive factors could be identified on multivariable analysis (see online resource Table 3). Changes from ISUP at cancer relapse after FT in comparison with S-RP specimen are shown in Fig. 2b.

### Oncological outcomes

**Fig. 3:**
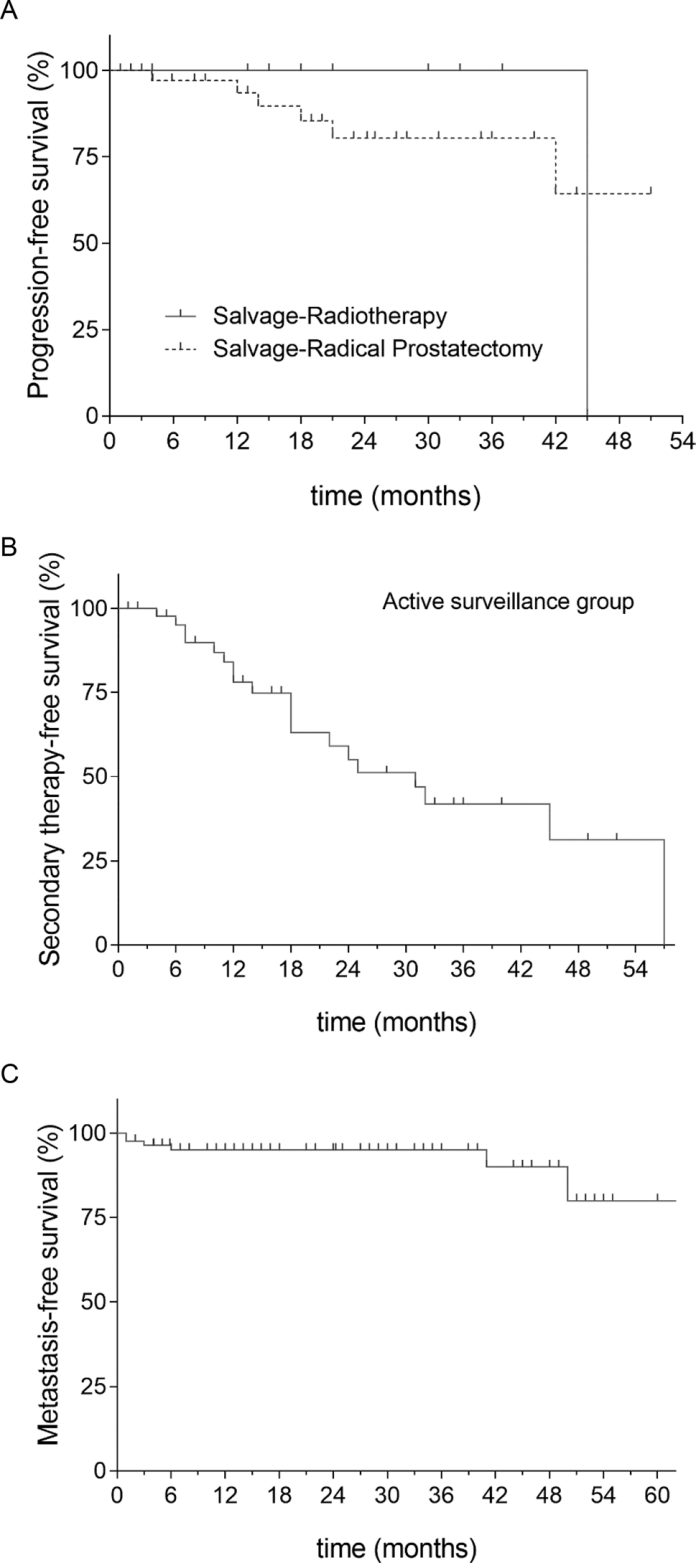
Kaplan–Meier estimates of **a** patients experiencing progression after salvage radical prostatectomy (S-RP) and salvage radiotherapy (S-RT), **b** patients under active survaillance with secondary-therapy during the follow-up period. **c** metastasis-free survival in the whole cohort. One patient in the S-RP group with no BCR and a follow-up of 98 months is not shown in **a** and **c**

Median (IQR) follow-up were 28 months (15–44.5) in the S-RP group [S-RT: 34.5 (26.3–46.3); AS: 19 (8.3–36)]. Figure 3a shows the estimates of PFS after salvage therapy in the S-RP and S-RT groups. 80.4% of the men in the S-RP group and 100% of men in the S-RT group experienced PFS at three years. Of the 50 men in the AS group, 41.7% received no secondary salvage therapy by S-RP, S-RT, ADT or Re-FT at three years, shown in Fig. 3b. In the whole cohort, 80% of the patients were estimated metastasis-free at five years (Fig. 3c). Evidence of metastases included the presence of lymph-node involvement (four men at S-RP) and distant metastases (one man at each the S-RP and the S-RT group).

## Discussion

We report the first multi-center analysis with comparative presentation of triggers and outcomes after S-RP, S-RT and AS in patients with cancer relapse who underwent FT. AS patients were carefully selected indicated by the lowest PSA levels, ISUP groups, percent of positive biopsy cores and percent of maximal cancer core infiltration compared to the S-RP and S-RT groups. Despite the up-to-date diagnostic workup APF at S-RP specimens could not be predicted. Estimated effects indicate an important role of ISUP at relapse after FT. Progression-free survival probability after S-RP and S-RT was sufficient at short to intermediate-term follow-up. Differently, in patients undergoing AS the secondary-therapy-free probability was low at short to intermediate-term follow-up.

Patients initially choosing FT make a trade-off that prioritizes quality of life and most patients do not regret this path as could be shown in one of our earlier studies [9]. Regret is higher in patients that experience cancer relapse after FT (OR 12.3). To prevent from further regret predictors for APF at S-RP specimen could serve as a basis for decision-making of an oncologic safe salvage therapy. Combined MRI-targeted and systematic biopsy in primary prostate cancer diagnosis have been shown to lead to a marginal rate of upgrading to ISUP GG 3 or higher (3.5%) at radical prostatectomy specimen [10]. Probably due to ablation energy defects after FT and often invisible lesions on MRI, the rate of upgrading on final pathology was high in our cohort even with most patients receiving a MRI-targeted and systematic fusion biopsy. ISUP at relapse after FT was associated with APF, but a prediction model could not be generated. This hampers patient counseling. A prediction model for biochemical recurrence on the basis of S-RP specimen identified infield recurrence (HR 3.77) and pT3b stage (HR 5.0) as most relevant risk factors [11]. Both factors are difficult to safely identify on the basis of control biopsy core location and MRI.

S-RP after FT has been explored previously: despite a similar ISUP distribution at cancer relapse after FT to the reported series of 82 men by Marconi et al. [11] our 3 year PFS was higher (80.4 vs. 36%). This could be explained by the early diagnosis of relapse after FT in our cohort and subsequent early S-RP at a median of 15 months compared to 26.5 months. A poor prognosis in terms of biochemical control was also demonstrated after S-RP with a 56.3% recurrence probability at 2 years [12] and the late diagnosis after FT (median of 24 months) discussed as the potential reason. Another study supporting this thesis performed S-RP in 34 men after FT at a median of 10.4 months and reported a biochemical-free survival of 79.4% at 4 years [13] similar to our observed outcome.

Despite the initial low PSA levels and mostly favorable histopathological findings in the AS group, the secondary-treatment rate seems high in our cohort. We cannot rule out that patient anxiety but not disease-related factors were relevant for conversion to secondary treatment. The high rate of secondary treatment in the AS group suggests that any cancer on control biopsy should be reported in FT trials as any cancer might lead to further salvage treatments. Despite the high secondary-treatment rate many patients circumvent early whole-gland treatment with potential side effects. This advantage should not be neglected.

The general challenge in prostate cancer to identify patients for AS also applies for cancer relapse after FT. How can we identify the optimal AS candidates with avoidance of overtreatment but without compromising the window for cure in this scenario? A further difficulty is that PI-RADS v2.1 scoring system is designed for treatment-naïve prostates and no scores for the ablation zone after FT have been widely adopted [14]. Nevertheless, AS protocols integrating mpMRI as a radiological biomarker in the untreated zone and ignoring the number of positive cores might improve AS for patients after FT. Such a protocol has been suggested by Alberts et al.: men with ISUP 1, PI-RADS score ≤ 3 and low PSA density < 0.15 ng/ml^2^ have shown no upgrading during AS [15]. These patients could be AS candidates after detection of cancer relapse after FT. A consensus statement on surveillance after prostate FT even suggests not to further treat cancer relapse ISUP 2 in the treated zone if < 0.2 cc or < 7 mm [16]. Our data from S-RP specimen show that the final ISUP is hardly predictable from combined systematic and targeted biopsy, which questions this approach.

The efficacy of S-RT after FT has been described in 21 patients in one report so far [7]. The median time until detection of cancer relapse after FT was longer (32 months) and PSA levels lower (4.6 ng/ml). None of the patients experienced biochemical recurrence or metastatic disease at three years which is similar to our report. Nevertheless, both series are too small to draw conclusions regarding the impact of prior FT on the outcome of S-RT. Furthermore, keeping in mind that FT of the peripheral zone harbors the risk of periprostatic adhesions. This might increase the toxicity of S-RT: it was described after whole-gland HIFU after less than three years of follow-up [17].

Our study is limited by its retrospective design. Second, the collection of data does not balance the potential bias from treatment indication patterns from each center. The further caveat in FT is the limited number of treated patients and hence an even smaller number of patients that underwent salvage therapies. This can be observed across several studies in the field and limits the potential of statistical analysis. Localization of cancer relapse could only be provided on the basis of biopsy documentation and not on whole-mount histology after prostatectomy and a case by case review by an uropathologist and the treating urologist, which we consider as gold standard for accurate evalutation.

## Conclusions

In clinical practice early control biopsy after FT as recommended by several consensus statements [18] to detect potential cancer relapse seems a key to sufficient oncologic control at short to intermediate follow-up after S-RP and S-RT. AS could postpone further treatment in a significant proportion of patients, but a high secondary-therapy rate at intermediate follow-up was observed. Further comparative studies are warranted to better choose an individualized risk-adaptive salvage therapy.

## Supplementary Information

Below is the link to the electronic supplementary material.

Supplementary file1 (DOCX 17 KB)

Supplementary file2 (DOCX 34 KB)

## Data Availability

Not applicable.

## Data availability

The data that support the findings of the study are available from the corresponding author upon reasonable request.
