# Supplementary material for: Triggers and oncologic outcome of salvage radical prostatectomy, salvage radiotherapy and active surveillance after focal therapy of prostate cancer
Source: World J Urol. 2021 Apr 21;39(10):3747–54. doi: 10.1007/s00345-021-03700-x (PMC8519844; doi:10.1007/s00345-021-03700-x)
Supplement: Supplementary file 1 — Supplementary file1 (DOCX 17 KB) [file 345_2021_3700_MOESM1_ESM.docx]

**Supplementary Table 1:** D´Amico risk classification of patients before focal therapy (FT) and at cancer relapse (CR) after focal therapy.

| D´Amico Risk Group | S-RP Group | | S-RT Group | | AS Group | |
| --- | --- | --- | --- | --- | --- | --- |
|  | FT | CR | FT | CR | FT | CR |
|  | n (%) | | | | | |
| Low | 20 (45.6) | 13 (29.5) | 6 (46.2) | 2 (15.4) | 20 (40) | 38 (76) |
| Intermediate | 13 (29.5) | 22 (50) | 7 (53.9) | 8 (61.5) | 23 (46) | 11 (22) |
| High | 2 (4.5) | 9 (20.5) | 0 | 2 (15.4) | 1 (2) | 0 |
| Missing | 9 (20.5) | 0 | 0 | 1 (7.7) | 6 (12) | 1 (2) |

**Supplementary Table 2:** Results of univariable logistic regression model: Predictors of adverse pathological findings at salvage radical prostatectomy (S-RP) specimen

|  |  | univariable analyses | | |
| --- | --- | --- | --- | --- |
| Variable | Direction and Unit | *p* Value | Odds Ratio | 95.0 % CI |
| Age at S-RP | Continuous (years) | 0.786 | 0.990 | 0.917 - 1.067 |
| PSA at S-RP | Continuous (ng/ml) | 0.153 | 1.120 | 0.974 – 1.332 |
| ISUP at cancer relapse | Categorical | 0.006 | 2.323 | 1.346 – 4.593 |
| Systematic biopsy cores positive at cancer relapse | Continuous (%) | 0.320 | 1.021 | 0.981 – 1.066 |
| Max. infiltration of cores at cancer relapse | Continuous (%) | 0.073 | 1.000 | 1.000 – 1.070 |
| PI-RADS on mpMRI at cancer relapse | Categorical | 0.161 | 1.250 | 0.920 – 1.727 |
| PSA change Nadir-S-RP | Continuous (%) | 0.189 | 1.006 | 0.998 – 1.017 |
| PSA velocity after Nadir | Continuous (ng/ml/y) | 0.585 | 0.923 | 0.677 – 1.232 |
| Time focal therapy to salvage therapy | Continuous (months) | 0.672 | 0.991 | 0.948 – 1.034 |

CI: 95% confidence interval for OR

**Supplementary Table 3**: Results of multivariable logistic regression model: Predictors of adverse pathological findings in S-RP specimen

|  |  | multivariable analyses | | |
| --- | --- | --- | --- | --- |
| Variable | Direction and Unit | *p* Value | Odds Ratio | 95 % CI |
| Age at S-RP | Continuous (years) | 0.758 | 0.981 | 0.864 - 1.110 |
| PSA at S-RP | Continuous (ng/ml) | 0.889 | 1.017 | 0.800 – 1.292 |
| ISUP at cancer relapse | Categorical | 0.066 | 1.965 | 1.018 – 4.534 |
| Max. infiltration of cores at cancer relapse | Continuous (%) | 0.187 | 1.028 | 0.988 – 1.078 |
| PI-RADS on mpMRI at cancer relapse | Categorical | 0.310 | 1.233 | 0.831 – 1.901 |
| PSA change Nadir-S-RP | Continuous (%) | 0.489 | 1.004 | 0.992 – 1.017 |

The multivariable model included age and all variables with p<0.2 at univariable analyses. CI: 95% confidence interval for OR
