# Supplementary material for: Triggers and oncologic outcome of salvage radical prostatectomy, salvage radiotherapy and active surveillance after focal therapy of prostate cancer
Source: World J Urol. 2021 Apr 21;39(10):3747–54. doi: 10.1007/s00345-021-03700-x (PMC8519844; doi:10.1007/s00345-021-03700-x)
Supplement: Supplementary file 2 — Supplementary file2 (DOCX 34 KB) [file 345_2021_3700_MOESM2_ESM.docx]

**Supplementary Figure 1:** Consolidated Standards of Reporting Trials (CONSORT) diagram of study inclusion.

Patients with proven cancer relapse after

focal therapy (FT) of localized prostate cancer (n=98)

8 excluded

1 initiation of long term androgen deprivation only

3 FT not for primary PCa

4 Repeat-FT

.

Salvage radiatiotherapy

(S-RT) (n=3)

Salvage radical prostatectomy

(S-RP) (n=37)

Active surveillance (≥ 6 months) (n=50)

Secondary S-RP (n=7)

Secondary S-RT (n=10)
